# Section on Ophthalmology, Otology and Laryngology

**Published:** 1881-06

**Authors:** 


FIRST DAY.

OPHTHALMOLOGY, OTOLOGY AND LARYNGOLOGY.—Chairman, Dr. D. S. Reynolds, of Kentucky; Secretary, Dr. S. M. Burnett, District of Columbia.

A paper was read by Dr. G. T. Stephens on the *Perimeter*, invented by himself for measuring the field of vision. Dr.

Stephens described the instrument closely, remarking that most of the instruments hitherto used are very large, weighing a good many pounds, and consequently inconvenient for using. His instrument removes some of the difficulties. The gentlemen present were invited to examine the instrument.

Dr. Wilson, of Philadelphia, wished to know whether Dr. Stephens' method was superior to the old plan, and whether any comparative measurements had been made. Dr. Stephens said he had discontinued the old methods, and was thoroughly convinced of its efficacy, but that no measurements had been made.

Dr. W. C. Jarvis next addressed the meeting on the subject of *Nasal Catarrh with Hypertrophy.* He remarked that the persistence of the disease is due to hypertrophy of tissue. This must be removed, and instruments are necessary to effect this result. The écraseur is the same instrument described in the *Medical Record*, April, 1881. The wire used is very fine, but exceedingly strong, and made of the best steel. The transfixion needles are provided with metal handles, and are made of various sizes. The simpler forms of hypertrophies can be removed with the écraseur alone, but sessile growths require both. Dr. Jarvis stated that all degrees of hypertrophy can be removed by means of these instruments. In one patient who had not breathed through the nose for fifteen years, and in one who had not breathed at all through the nostril, the relief was almost immediate. All kinds of nasal growths can be reached by means of the instruments, and there is no longer any use of resorting to caustics, cutting and the galvano-cautery.

Dr. Smith, of Detroit, wished to know if nasal respirations were immediate, and also how long a mechanical trouble could last without producing organic change.

Dr. Jarvis stated that he was simply stating the fact as it stood; that the patient was not completely deaf, but that he had to be spoken to in a loud tone of voice.

Dr. Jos. A. White said that he had used the instrument mentioned by Dr. Jarvis, and had obtained excellent results. In one case in which the hearing was only $\frac{2}{50}$, the result was such as to produce perfect hearing on one side, and partial hearing on the other side, where organic trouble was added.

Dr. Daly said that the instrument was a very useful one. In his experience, deafness of greater or less degree has often been relieved by removal of nasal growths, and that, too, without treating the deafness at all. If the "central trouble" or hypertrophy be remedied, the result will be good. The galvano-cautery, used for a few moments at a time, is an excellent means of removing the growth in some instances. The finger is introduced into the pharynx as a support and protection. In some cases glacial acetic acid answers well as an application to the stump after the removal of the growth with instruments. Cases under his care have been watched for years, and have progressed favorably. In one case in which sessile growths were present, these were removed with the galvano-cautery, heated to a mahogany or cherry red. The apex of the growth disappears, gradually becoming hollowed out and sound, normal structure is left. The surrounding structures are not injured.

Dr. Rigsby wished to know to what extent deafness is due to anterior nasal catarrh.

Dr. Jarvis said that the extent was undetermined, but that the pharyngeal trouble is secondary to the anterior disease. The acrid secretions of the part flow backward and irritate the healthy mucous membrane. A mild astringent injection may sometimes give rise to the pharyngeal disease, and it is highly probable that the secretions of the part diseased can act in the same way.

Dr. Rigsby said he had been struck, after a long experience, with the connection between aural and nasal diseases. Deformities in the vomer and turbinated bones produce trouble in the ear, after giving rise to hypertrophy and sometimes ulceration in the nose. Deflection of the vomer to one side is one deformity giving rise to ear disease. Operative interference is not often needed. Iodoform, with alum or acacia, is an excellent application. In other cases, where no deformity exists, the rhinoscope will reveal a group of enlarged glands. These give rise to a viscid secretion which irritates the surrounding structure.

Dr. Little, of Philadelphia, stated that we must not forget the effect upon the lachrymal canal of nasal troubles; it is an important one.

The chairman remarked that the predisposing causes are very important, but had not been discussed. Do cases of cured catarrh stay well? and is not the local trouble secondary to constitutional causes?

Dr. Little remarked that traumatic causes must be considered. He had seen a child strike its nose and have catarrh. This occurred in a healthy person.

Dr. Jarvis then said that two causes of catarrh must be considered—a local deformity or hypertrophy, and an atonic condition of the system. A distinction should be made between temporary and permanent stenosis. Deflection of the vomer always causes catarrh. These cases get worse as the patient grows older. In some cases the bones have been crushed, but this treatment is to be condemned. The instrument exhibited by himself would, he thought, answer every purpose.

Dr. Carter said that one great cause seemed to him to be the change from the impure hot air of a room to the cold air without, and advises his patients to place a piece of cotton in the nostril on going into the open air. The oxide of mercury he has used with a great deal of benefit. The great point is the recurrence of the trouble, and it does frequently recur.

Dr. Daly thinks that a fair proportion of cases depend upon a constitutional defect, but many of those are pharyngeal catarrh. Subjects with slow and bad digestions are liable to this form. A smaller per cent. of nasal troubles are constitutional. The majority are in excellent health, and we must look upon the nasal disease as the result of repeated colds in the head. In such cases, temporary stenosis takes place; this is relieved, but a slight thickening is left, which is increased by each subsequent attack. Subsequently nervous tone is impaired and vascular action interrupted. The best way to deal with the patient is to tell him that the trouble can be cured, but that it will recur if he catches cold again. Of course he should consult a physician, in that case, and be treated again. Dr. Daly has often seen the constitution improve after local relief.

Dr. Stephens said that the gentleman was mistaken, he thought, if the majority of these cases was referred to local causes; certain people are predisposed to colds and catarrh. Why is it? We must try to find out. Reflex irritation, as for instance, uterine disease may predispose to catarrh. An accumulation of cerumen in the ear, or diseases of the eye, can produce the same result. All such causes should be removed.

Dr. Chisholm then read a paper on the Treatment of Conical Cornea. He said that it is a pertinent question how to get the projection back. Suggestions had been made of cutting out a piece of the cornea and bringing the surfaces together. After reading a French article on puncture of the cornea with a needle heated to redness, he determined to put it into practice. A case of exophthalmic goitre with a complication of eye troubles was selected; objects could not be distinguished one foot off. No anæsthetic was given and the needle was thrust suddenly through the cornea. The anterior chamber was immediately emptied; no pain was felt; and atropia and cold water were used. There was no inflammatory action. As soon as the eyes were opened the patient read at six feet. The same operation was used on the right eye, and eight months afterward the cornea was perceptibly flattened—sufficiently so as to admit of an iridectomy.

Dr. Little said that he had assisted Dr. Noyes, of New York, in a case of conical cornea, where tapping was resorted to. The result was highly satisfactory.

### SECOND DAY.

OPHTHALMOLOGY, OTOLOGY AND LARYNGOLOGY.—Chairman, Dr. D. S. Reynolds, of Kentucky; Secretary, Dr. S. M. Burnett, District of Columbia, met at Wilkinson's Hall, at 3 o'clock P. M.

The Section was called to order by the Chairman, and the minutes of the previous meeting were read by the Secretary.

The first paper read was upon the subject of Syphilitic Laryngitis, by Dr. Carl Seiler, of Philadelphia, in which he stated that the affection could be diagnosed from non-specific inflammation by the peculiar discoloration of the mucous membrane and the symmetrical disposition of the inflammatory patches. There are frequent ulcerations of the larynx, which may be divided into shallow ulcers, in nothing differing from those seen in catarrhal laryngitis, and deep ulcerations, which were, in the author's opinion, due to the breaking down of the smaller or larger gummata in the mucous membrane. He also stated that a diagnostic sign of syphilitic laryngitis was seen in the red lines observed upon the velum palati. He recommended as treatment, beside the systemic, with iodide of potassium and mercury, and supportive, with tonics, etc., local touching of the shallow ulcers with solid nitrate of silver fused upon an aluminum probe, and the deep ulceration with acid nitrate of mercury (1 to 4), or the galvanic cautery.

The subject being open for discussion, Dr. Reynolds said that he thought constitutional treatment would do more good than any local measures. He was surprised at the statement of Dr. Seiler, that the diagnosis of simple form of syphilitic laryngitis was difficult. He did not believe that catarrhal inflammation could produce ulceration. There were three kinds of syphilitic ulcers, viz.:

1. The ulcer with inflamed base and everted edges.

2. Pale or gray base with irregular margin and sharply defined scarlet areola.

3. Ulcer wholly unaccompanied with any evidences of inflammation with sharply defined outlines and gummatous deposits surrounding the margin.

He thought that local applications could give no good results, unless restricted to such remedies as would give relief from pain. While not doubting the correctness of Dr. Seiler's observations, he differed with him in his diagnosis.

Dr. Stephens, of Hartford, thought the treatment much more satisfactory when both local and constitutional remedies were employed. He used nitrate of mercury (1-4) locally, with the internal administration of potass. iodide.

Dr. Walsh said that he thought too much prominence was given to the iodide of potassium. He held the opinion that the shallow ulcer corresponded in its time of appearance with the mucous patch of syphilis, and the deeper ulceration with the tertiary stage. He always found enlargement of the cervical glands in specific laryngitis. He employed mercury internally, especially in the shallow ulceration, and iodide of potassium in the deeper forms. He thought Dr. Reynolds wrong in neglecting local medication, the combination of the local and constitutional treatment giving better results. Of the modes of administering mercury, he preferred inunction. Treatment should be supporting. Dr. Seiler closed the discussion, saying he thought catarrhal inflammation could produce ulceration. The infiltration of the sub-mucous tissue could break down and produce ulceration. In his paper he had taken it for granted that any physician would employ constitutional treatment in this disease. He claimed that local treatment lessened the amount of cicatricial contraction in the process of cure. The application of caustic served to coat over the ulcer and protect it. He claimed that there was involvement of the cervical glands often in non-specific laryngitis, while it was not a constant symptom in the specific form. He based his diagnosis mainly upon the carmine color of the mucous membrane and the symmetrical arrangement of the ulcers. If there was an ulcer upon one side of the larynx, there would be found a corresponding ulcer or focus of inflammation on the other side. He attached much importance to the red lines upon the velum palati, which were sometimes brought out by the irritation of such an examination.

Dr. Chisholm read a paper on a form of tinnitus induced by a rhythmical contraction of the tensor tympani muscle, which was first brought to his notice by a muscular twitching in his own left ear. It occurred during the heat of summer, and invariably commenced after dinner. The twitching at first was not of more than half an hour's duration. It did not affect the hearing. The next day after dinner the buzzing sound was resumed. This followed for four days in succession. The duration of the buzzing now was gradually increased, until the last recurrence. The noise continued until lost at bed-time in sleep. Inflation of air, chloroform vapor, electricity, both Faradic and continuous, did not in any way control the buzzing sound. The fluttering was felt at the drum head, and analyzed as a rhythmical contraction of the tensor tympani muscle. The contraction and relaxation varied at times from 130 to 160 to the minute. The cause was ultimately found to be a glass of wine taken at dinner, in connection with the very hot weather. Omission of the wine stopped the noise, and its resumption at dinner renewed it. Other cases of a similar nature have come under the observation of Dr. Chisholm. The muscular twitching was similar to the twitchings so often experienced in the muscular fibers of the eye-lid, and which may be kept up for minutes, hours, or even days. There was no diminution of the hearing power, even while the noisy muscular contraction was greatest.

Dr. Burnett had noted in his own case that when he made a strong contraction of his orbicularis muscle there was a peculiar sound in the ear of the same side. He at first thought it was the sound of muscular contraction conveyed through the bones of the head, but this was disproved by the fact that he could only produce the sound at certain times. He could always do it in the morning. He attributed it to contraction of the tensor tympani muscle.

Dr. Stephens had a patient suffering with tinnitus, who came to this country for change of climate, but received no benefit from it. He used the ordinary treatment without success. Hydrobromic acid, which usually gave good results, did no good in this case.

Dr. Eugene Smith, of Detroit, read a paper describing a successful operation for blepheroplasty, in which the graft was taken from the arm without any pedicle. A piece of skin, one and a half by two inches, was taken from the arm, and the cellular tissue and a portion of the true skin shaved off. This was then applied to the cut surfaces of the lid, being about one-fourth larger than the wound. The case progressed to a very successful termination, the motions of opening and closing the eye being perfect. Dr. S. exhibited photographs of the patient before and after the operation. There had been no contraction of the lid for several months. The operation was performed in October last.

Dr. H. Augustus Wilson, of Philadelphia, reported a case operated upon by Dr. Levis, in which the graft progressed well for two weeks, and then sloughed, leaving the condition as bad as before operation. The skin of an amputated little finger was used in this case, but the cellular tissue was not removed. He attributed the failure to this fact.

Dr. Burnett mentioned a case where the contraction afterwards was continuous, until it did away with all the good results of the operation. He thought this was the most frequent cause of failure.

Dr. Smith said he was thus particular in describing the operation, because he had twice before failed in the same. He attached much importance to shaving away all of the cellular tissue and a portion of the cutis vera, thus bringing the dermic cells in direct contact with the cut surface. He thought failure was generally due to want of care in preparation of the graft. Flap should always be one-fourth to one-half larger than the surface to be covered.

Dr. Reynolds described a similar operation performed by him in 1862. First graft came away in the night, causing severe hæmorrhage. The second was successful, and the patient, a practicing physician, is now doing well.

Dr. Wilson, of Philadelphia, exhibited to the Section a lot of compressed hypodermic pellets, introduced to the profession by him some years ago. They were originally intended for solution for hypodermic use, to prevent keeping solutions for this purpose. He was in the habit of using one containing $\frac{1}{100}$ grain of atropia and $\frac{1}{4}$ grain sulphate sodium for relaxing the accommodation. He simply placed it in the conjunctival sac of the lower lid, and it was absorbed in about two minutes. It would relax the accommodation in an hour. One application was usually sufficient.

Dr. Risley, of Philadelphia, could not understand how so smal an amount could produce paralysis of accommodation in so short a time.

A long discussion was here indulged in, as to the amount of atropia necessary, and how often it should be repeated, to produce the required relaxation, and the means used to test the loss of accommodational power. Many and different views were set forth by as many gentlemen ; and with this discussion the meeting of the Section closed.

Just before adjournment, a paper by Dr. Lawrence Turnbull, of Philadelphia, entitled, (1) Otitis Intermittens, with Observations upon the use of Quinine in Diseases of the Ear ; (2) On the importance of Ear Examinations in effecting Life Insurance, was, on motion, read by title, and referred to the Committee on Publications.

